# Bilateral vestibulopathy decreases self-motion perception

**DOI:** 10.1007/s00415-021-10695-3

**Published:** 2021-07-14

**Authors:** Lisa van Stiphout, Florence Lucieer, Maksim Pleshkov, Vincent Van Rompaey, Josine Widdershoven, Nils Guinand, Angélica Pérez Fornos, Herman Kingma, Raymond van de Berg

**Affiliations:** 1 Department of Otorhinolaryngology and Head and Neck Surgery, Division of Balance Disorders, School for Mental Health and Neuroscience, Maastricht University Medical Center, P. Debyelaan 25, 6229 HX Maastricht, The Netherlands; 2 Faculty of Physics, Tomsk State Research University, Tomsk, Russian Federation; 3 Department of Otorhinolaryngology and Head and Neck Surgery, Faculty of Medicine and Health Sciences, Antwerp University Hospital, University of Antwerp, Antwerp, Belgium; 4 Service of Otorhinolaryngology Head and Neck Surgery, Department of Clinical Neurosciences, Geneva University Hospitals, Geneva, Switzerland

**Keywords:** Bilateral vestibulopathy, Perceptual self-motion thresholds, Vestibular, Perception, Threshold, Self-motion perception

## Abstract

**Objective:**

Current diagnostic criteria for bilateral vestibulopathy (BV) primarily involve measurements of vestibular reflexes. Perceptual self-motion thresholds however, are not routinely measured and their clinical value in this specific population is not yet fully determined. Objectives of this study were (1) to compare perceptual self-motion thresholds between BV patients and control subjects, and (2) to explore patterns of self-motion perception performance and vestibular function in BV patients.

**Methods:**

Thirty-seven BV patients and 34 control subjects were included in this study. Perceptual self-motion thresholds were measured in both groups using a CAREN platform (Motek Medical BV, Amsterdam, The Netherlands). Vestibular function was evaluated (only in BV patients) by the caloric test, torsion swing test, video head impulse test of all semicircular canals, and cervical- and ocular vestibular-evoked myogenic potentials. Differences in thresholds between both groups were analyzed. Hierarchical cluster analysis was performed to visualize patterns between self-motion perception and vestibular function within the group of BV patients.

**Results:**

Perceptual self-motion thresholds were significantly higher in BV patients compared to control subjects, regarding nearly all rotations and translations (depending on the age group) (*p* ≤ 0.001). Cluster analysis showed that within the group of BV patients, higher perceptual self-motion thresholds were generally associated with lower vestibular test results (significant for yaw rotation, caloric test, torsion swing test, and video head impulse test (*p* ≤ 0.001)).

**Conclusion:**

Self-motion perception is significantly decreased in BV patients compared to control subjects regarding nearly all rotations and translations. Furthermore, decreased self-motion perception is generally associated with lower residual vestibular function in BV patients.

**Trial registration:**

Trial registration number NL52768.068.15/METC

**Supplementary Information:**

The online version contains supplementary material available at 10.1007/s00415-021-10695-3.

## Introduction

Bilateral vestibulopathy (BV) is a chronic disorder in which the vestibular function is bilaterally reduced or absent due to deficits of the labyrinths, the vestibular nerves, the brain, or a combination of the above [1, 2]. Symptoms include unsteadiness when walking or standing, movement-induced blurred vision (oscillopsia) and unsteadiness worsening in darkness or on uneven ground. These symptoms, as well as documented bilaterally reduced or absent angular vestibular-ocular-reflex (VOR) function, are used as diagnostic criteria [3]. Additional symptoms can also be present, like depression, anxiety, cognitive impairment and the increased risk of falling. This can lead to a deterioration of quality of life and might have a strong socio-economic impact [4–8]. Reported prevalences vary from 28 to 81 per 100,000 people, and at this moment, there is no evidence of an effective medical treatment to restore vestibular function [5, 9, 10].

Since BV is a heterogeneous chronic disorder with different involvement of the vestibular system, it can present with various clinical pictures. Therefore, it poses a diagnostic challenge, as different outcomes from diagnostic tests can be found, such as a falsely normal clinical Head Impulse Test (because of compensatory covert saccades) and normal rotatory chair test results [2, 11–15]. For this reason, it is often mis- or underdiagnosed [15, 16].

Current diagnostic tests included in the criteria for BV primarily measure reflexes (e.g. caloric test, video Head Impulse Test (vHIT), and torsion swing test) [3]. In contrast, perceptual self-motion thresholds are not routinely measured. This might be complementary, since approximately one-third of patients with vestibular complaints have normal results on the routine diagnostic tests that measure reflexes [17]. Furthermore, perception of self-motion involves a network of various regions and structures in the cortex (e.g. the ventral intraparietal area, the parieto-insular vestibular cortex and the medial superior temporal area), the cerebellum (e.g. the nodulus/uvula and the fastigial nucleus), the thalamus (e.g. ventroposterior complex), and brainstem (vestibular nuclei in the dorsolateral pons and medulla) [18]. This explains why, for example, perceptual thresholds for head motion can be elevated in patients with absent cerebellar function [19].

Perceptual thresholds can be measured using different testing paradigms, such as extensive research-oriented protocols [11, 17, 20–24] or a new and more clinically oriented and faster testing protocol [25]. These testing paradigms are similar in ways that: the provided stimuli are determined by the subject’s responses on previous trials (adaptive approach) [26]; the subject is not allowed to make an indifferent response (i.e. the subject is instructed to guess when he/she is uncertain about the perceived stimulus: forced-choice); a single stimulus is provided (one-interval paradigm); and the subject needs to discriminate between a positive or a negative stimulus (e.g. a rightward from a leftward motion: recognition task) [22]. The differences between extensive research-oriented protocols and the more clinically oriented testing protocol are the type of stimulus used and the amount and type of motion-directions tested. Regarding type of stimulus, the more lengthy, research-oriented tests use a fixed frequency with sinusoidally shaped acceleration profiles. The clinically oriented testing paradigm is based on stimuli with the longest possible exposition to constant peak acceleration (plateau phase), which might be considered the main stimulus parameter of interest. However, this implies that these stimuli are not frequency-fixed, due to limitations of the platform. Regarding amount and type of motion-directions tested, research-oriented protocols operate with two-option paradigms, whereas the clinically oriented protocol operates with a twelve-option paradigm. One-interval recognition two-option tasks used in research-oriented protocols for detecting self-motion perception as a function of frequency, have a duration of three up to 12 h of testing [11, 20, 22, 23], while the more clinically oriented and twelve-option testing paradigm has a maximum duration of one hour [25]. The clinically oriented test is potentially easier to translate to the clinical practice and might be more applicable for patients with BV since vestibular loss negatively affects attention performance [27].

Abnormally elevated perceptual self-motion thresholds were previously reported in patients with BV using extensive research-oriented protocols [11, 17, 23, 24, 28]. Diverse outcomes were reported regarding the affected type of motion, possibly indicating the relative sparing of different sensors of the vestibular system [11, 23, 24, 29–31]. Although research showed that BV is a heterogeneous disorder with different involvement of the vestibular system [12, 13, 15, 32], patterns of vestibular function and self-motion perception performance evaluated with the clinically oriented testing paradigm have not yet been explored.

The clinical value of testing perception of self-motion in a BV population is not yet fully determined [17, 25]. However, it might be used as one of the outcome measures of rehabilitation, for example to evaluate the functional effect of vestibular implantation [33–36]. This would be in line with research involving noisy galvanic vestibular stimulation, which showed that noisy galvanic stimulation is able to improve self-motion perception in healthy subjects and patients with BV, next to improving postural control and gait performance, in research settings [37–47].

Taken all the existing evidence regarding self-motion perception in BV patients into account, it might be beneficial to investigate these aspects in a relatively larger patient cohort using the less elaborate and more clinically oriented testing paradigm, complemented with detailed clinical data concerning vestibular function. Therefore, objectives of this study were (1) to evaluate the effect of vestibular input on self-motion perception by comparing perceptual self-motion thresholds tested with the clinically oriented and faster testing paradigm between a relatively large cohort of BV patients and control subjects, and (2) to explore patterns of self-motion perception performance and vestibular function in patients with BV. It was hypothesized that patients with BV have higher self-motion perception thresholds compared with control subjects, tested with the clinically oriented testing paradigm. Next to this, it was hypothesized that patterns would emerge showing (a) lower self-motion perception performance regarding rotations combined with an absent or reduced function of the semicircular canals, and (b) lower self-motion perception performance regarding translations combined with dysfunction of the otolith organs [11, 14, 15, 48].

## Methods

### Subjects

Thirty-seven patients with BV diagnosed at Maastricht University Medical Center, were included in this study [mean age 60 years (range 42–79 years); 18 females (48.6%)]. Diagnostic criteria for BV included imbalance and/or oscillopsia during walking or head movements, and a reduced bithermal caloric response (sum of bithermal maximal peak slow-phase velocity < 6°/s bilaterally) and/or a bilaterally reduced vHIT gain of < 0.6, and/or a VOR gain < 0.1 during torsion swing test. Subjects with polyneuropathy and subjects who were not able to stop vestibulo-suppressive medication, sit in the testing chair for one hour, or unwilling to undergo one of the detailed physical or vestibular examinations, were excluded from participation in this study. All subjects were tested by the same examiner (FL).

The control group comprised 34 control subjects, with a mean age 61 years (range 42–77), of which 21 were female (61.8%) (previously described by Dupuits et al. [25]). Subjects were excluded in case of current (or history of) vestibular disease, migraine, use of vestibulo-suppressive medication, or an inability to take place in the testing chair for at least one hour.

### Perception platform

Perceptual self-motion thresholds were measured in both groups: control subjects and BV patients. Testing perceptual self-motion thresholds has been previously described [25]. In summary, a hydraulic CAREN platform combined with D-flow 3.22.0 software (Motek Medical BV, Amsterdam, The Netherlands) was used to measure the perceptual self-motion thresholds within each subject. A 12-option paradigm, 6 translations and 6 rotations, was delivered by the platform. The subject sat in complete darkness in a chair mounted on the platform, fastened by a seatbelt, while wearing a blindfold and headset to mask visual and auditive cues. The six translations included motions in the horizontal plane (forward, backward, right, left) and in the vertical plane (up and down). The rotations included yaw left, yaw right, pitch forward, pitch backward, roll left and roll right. The stimulus profile was the same for each of the 12 different motions and it was composed of a smoothly increasing acceleration phase (low jerk) until a constant acceleration was obtained for a fixed duration (plateau phase), followed by a smooth decrease of acceleration (low jerk) down to zero. The non-linear parts of the stimulus were sinusoidal and the amplitude and frequency (the sine parameters) depended on the magnitudes of acceleration (a) and jerk (j), which varied for each separate motion stimulus. Concluding, every stimulus was controlled by three parameters: the maximum range, the acceleration magnitude, and the jerk magnitude.

Thresholds were found using an adaptive, forced-choice, one-interval direction-recognition paradigm with a 1-up-1-down staircase protocol with a maximum duration of 1 h. Motion trials started at the highest possible acceleration and their directions were randomly chosen by the examiner. After each motion, the subject was asked to inform the examiner about the type and direction of the perceived movement. In case the subject could indicate the correct type and direction of the movement, the stimulus was decreased with 0.1 m/s^2^ (translations) or 10 deg/s^2^ (rotations). In case of an incorrect response, the acceleration was increased with 0.05 m/s^2^ or 5 deg/s^2^. If the subject then could indicate the correct type and direction of movement, the acceleration was decreased with 0.03 m/s^2^ or 3 deg/s^2^. However, in case of an incorrect answer, the acceleration was increased with 0.03 m/s^2^ or 3 deg/s^2^. A double confirmation of the lowest threshold combined with double incorrect responses at the acceleration one step below threshold, was considered the perceptual self-motion threshold for that motion profile. The platform motion range was limited to 0.4 m for translational movements and 30° for rotational movements. Due to the physical limitations of the platform, the maximum and minimum measurable accelerations were, respectively, 0.4 m/s^2^ and 0.01 m/s^2^ for translations and 40°/s^2^ and 0.1°/s^2^ for rotations. A detailed illustration of the set-up and an overview of the twelve different motions can be found in Online Resource 1.

### Other vestibular tests

The caloric test, torsion swing test, vHIT, and Vestibular Evoked Myogenic Potentials (VEMPs) were only measured in patients with BV (see Online Resource 2 for exemplar data). All tests were performed by the same trained technician (FL).

#### The caloric test

The caloric test was performed using the Variotherm Plus device (Atmos Medizin Technik GmbH, Lenzkirch, Germany) in a completely dark room, with eye movement calibration performed before each irrigation. Subjects were positioned in a supine position with their head tilted 30° from the horizontal plane. Each irrigation lasted 30 s with a volume of at least 250 ml water for cold (30 °C) and warm (44 °C) irrigations in both ears. The first irrigation started with warm water in the right ear, followed by irrigation with warm water in the left ear. This procedure was followed by irrigation with cold water in the right ear, followed by irrigation with cold water in the left ear. A 5-min stimulus interval was kept between irrigations. Eye movements were recorded with electronystagmography (KingsLab 1.8.1, Maastricht University, Maastricht, The Netherlands). The self-adhesive electrodes (Blue sensor, Ambu, Denmark) were placed near the right and left exterior and interior canthi, above the eyebrows, on the orbital margin inferior to both eyes, and on the forehead.

#### Video Head Impulse Test

The horizontal vHIT and the vHIT in the Right-Anterior–Left-Posterior (RALP) and Left-Anterior–Right-Posterior (LARP) canal planes were performed using the Video-Head Impulse Test device from Otometrics (Otometrics, Taastrup, Denmark). The testing method was described previously [49]. In summary, the subject was sitting on a static chair to prevent body movements during the test. The technician stood behind the subject and held their head firmly without touching of the goggles. The subject maintained visual fixation on an earth-fixed target at a distance of 1.5 m. Head impulses comprised fast (peak velocity > 150°/s in horizontal plane, > 100°/s in RALP and LARP plane), unpredictable, low-amplitude (± 20°) head movements in the horizontal plane and in the RALP and LARP planes.

#### Torsion swing test

The torsion swing test was performed using sinusoidal rotation (0.1 Hz) with a peak velocity of 60°/s, while the subject sat in the chair (Ekida GmbH, Buggingen, Germany) in complete darkness. Eye movements were recorded with electronystagmography (KingsLab 1.8.1, Maastricht University, Maastricht, The Netherlands). The self-adhesive electrodes (Blue sensor, Ambu, Denmark) were placed near the right and left exterior and interior canthi, above the eyebrows, on the orbital margin inferior to both eyes, and on the forehead.

#### Vestibular Evoked Myogenic Potentials

Cervical VEMPs and ocular VEMPs were recorded as auditory-evoked myogenic potentials with the Neuro-Audio system with electromyographic software (v2010, Neurosoft, Ivanovo, Russia) and self-adhesive electrodes (Blue sensor, Ambu, Denmark). For cervical VEMPs, electrodes were placed on the belly of each sternocleidomastoid muscle. The reference electrode was placed on the sternum and a ground electrode was placed on the forehead. For ocular VEMPS, electrodes were placed on the orbital margin inferior to both eyes, and reference electrodes were positioned approximately 2 cm below them. An earth electrode was placed on the forehead.

Cervical VEMPs were measured over the sternocleidomastoid muscle after stimulating the ipsilateral vestibular organ with air-conducted tone bursts of 500 Hz, provided via inserted earphones at a stimulation rate of 13 Hz. Subjects were in a supine position with their back tilted in an angle of 30° from the horizontal plane and were asked to turn their head away from the location of the stimulus and to lift their head up slightly. A visual feedback system (v2010, Neurosoft, Ivanovo, Russia) connected with a monitor, showed the level of muscle contraction on a meter with green and red areas visible to the patient. The meter pointed towards the green area if muscle contraction was correct (between 65 and 205 µV) and was situated in one of the two red areas when muscle contraction was too low (below 65 µV) or too high (above 205 µV). The patient was instructed to keep the values of muscle contraction within the green area of the meter. Two-hundred electromyography (EMG) traces with a minimum rectified voltage of 65 µV and a maximum rectified voltage of 205 µV were accepted.

Ocular VEMPs were measured over the inferior oblique muscle after stimulating the contralateral vestibular organ with the same stimulation parameters as for cVEMPs. Subjects were in a supine position and were instructed to keep their eyes fixed on a focus point 30 degrees behind the head to achieve superomedial gaze. A minimum of 300 EMG traces were accepted.

VEMP thresholds were determined using a staircase approach with steps of 5 dB SPL, starting at 130 dB SPL. The sound level with an undetectable P1 and N1 peak just below threshold level was confirmed with a trial repetition. To reduce discomfort for the subjects, the number of trials was kept as low as possible.

### Data processing and analysis

#### Data collection and processing

Earlier studies on perceptual self-motion thresholds in healthy subjects reported different results with respect to the effect of age on translational and rotational thresholds [29, 50–53]. However, three recent studies found increased perceptual self-motion thresholds with age [20, 25, 54]. Since age might therefore significantly affect perceptual self-motion thresholds in healthy subjects [20, 24, 25, 54], both groups were split in age groups and the analysis of thresholds between control subjects and patients with BV was conducted for the age groups 40–59 years and 60–79 years.

Subjects who scored above the measurable threshold on the perception platform were rated with a threshold of 0.45 m/s^2^ for translations and 45°/s^2^ for rotations. Opposite movements in the same plane (e.g. yaw rotations to left and right) produced similar results and showed no statistically significant difference (see Online Resource 3 Table 1 for the Mann–Whitney *U* test statistics). Therefore, opposite movements in the same plane were combined and averaged. For the caloric test, the maximum peak slow-phase velocity at the culmination phase (°/s) was measured after each irrigation. For torsion swing test, VOR gain was calculated as peak eye velocity divided by peak head velocity. For the vHIT, VOR gain was calculated as the ratio of the area under the curves of eye velocity and head velocity. For cVEMPS, the lowest threshold with a present initial positive peak (at ± 13 ms) and a subsequent negative peak (at ± 23 ms) was documented. For oVEMPS, the lowest threshold with a present initial negative peak (at ± 10 ms) and a subsequent positive peak (at ± 15 ms) was documented. Two independent technicians determined the VEMP thresholds in consensus. Thresholds were categorized as present or absent (i.e. no present p13n23 or n10p15 wave at 130 dB SPL).

#### Statistical analysis

IBM SPSS Statistics version 25 and R version 3.5.2 were used for data analysis. Histograms, Q–Q plots and the Shapiro–Wilk test showed that the data were not normally distributed. Therefore, non-parametric analysis was applied to determine significant differences in thresholds between the control group and BV group and to determine significant differences in thresholds and results for tests of vestibular reflexes within BV clusters using the Mann–Whitney *U* test and medians [55]. *p* values below 0.05 were considered significant and were adjusted and reported with Bonferroni correction for multiple tests. Hierarchical cluster analysis was applied to explore and visualize patterns with respect to vestibular perception and tests of vestibular reflexes in patients with BV. Before clustering, the data of the BV group were standardized in *Z *scores (i.e. the individual scores minus the mean, divided by the standard deviation), to have the variables weigh equally in the cluster analysis. Age was not included as a variable in the cluster analysis since the effect of vestibular function was assessed, not the effect of age. Besides, no statistically significant difference was found in perceptual self-motion thresholds between the two age categories within the BV group (see Online Resource 3 Table 2 for the Mann–Whitney *U* test statistics). However, patient characteristics, such as age, were compared between clusters after performing the analysis. Ward’s method with the distance measure squared Euclidian distance was used, since Ward’s method has the highest agglomerative coefficient compared with the other hierarchical clustering methods. The silhouette method was used to determine the optimum number of clusters [56]. Hierarchical cluster analysis resulted in two dendrograms with patients on the *x*-axis and vestibular tests on the *y*-axis. A heatmap was created. Each column represented one subject, each row represented the output of a specific vestibular test. A “bad (vestibular) score” (i.e. high thresholds on platform tests and low scores on tests of vestibular reflexes) was illustrated by lower *Z* scores in the color red. A “relatively good (vestibular) score” (i.e. low thresholds on platform tests and relative high scores on tests of vestibular reflexes) was illustrated by higher *Z* scores in the color blue.

Because of the high inter- and intra-individual variability in absolute values of VEMP thresholds, VEMPs (left and right) were categorized as present or absent and separately analyzed. To analyze the relationship between present, present/absent or absent VEMPs and perceptual self-motion thresholds of translations, a Kendall’s tau-*b* correlation was ran.

## Results

### BV patients: relevant characteristics

In 54% of the included patients, a definite etiology could be determined, with ototoxicity as most common etiology. In the other 46%, the etiology was idiopathic or of probable cause. Eighty-nine percent of the patients had a bilaterally reduced caloric response (sum of maximal bithermal SPV < 6°/s in each ear), while 84% had a bilaterally reduced VOR gain measured with vHIT (< 0.6), and 60% had a reduced VOR gain on torsion swing test (< 0.1). Fifty-four percent of the patients met three of the criteria of the Bárány society described earlier, whereas 24% met two of the Bárány criteria and 22% only met one. A detailed overview of all relevant patient and control group characteristics can be found in Online Resource 3 Tables 3 and 4.

### Perceptual self-motion thresholds of BV patients compared to control subjects

All perceptual self-motion thresholds of translations could be determined in all control subjects, but not in all BV patients due to the physical limitations of the platform. Thresholds of translations in BV patients were above measurable threshold in 8% in the forward–backward plane, 22% in the left–right plane and 14% in the upward–downward plane. Regarding thresholds of rotations, also all thresholds could be determined in all control subjects, but not in all BV patients due to the physical limitations of the platform. These latter scored 43% above measurable threshold for yaw rotations, 41% for pitch rotations and 32% for roll rotations.

**Fig. 1:**
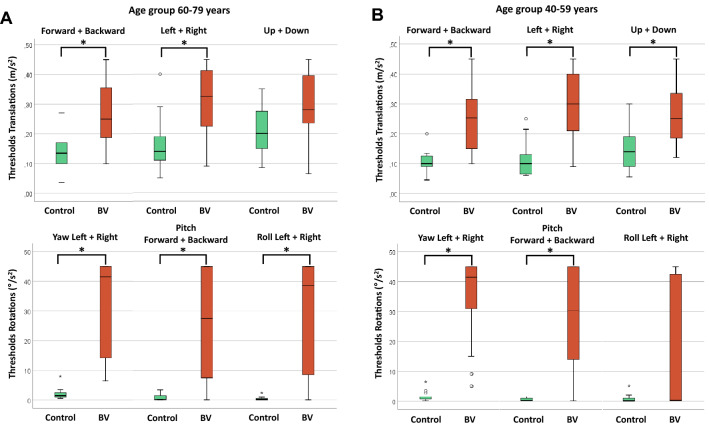
**a** Perceptual self-motion thresholds for each plane of movement, obtained in control subjects (*n* = 13) and patients with bilateral vestibulopathy (BV, *n* = 18), in the age group 40–59 years. Each box plot represents the 25–75 percentiles, whiskers indicate the 95 percentiles and bold black lines the median. Asterisks (*) illustrate statistically significant differences (*p* ≤ 0.001). **b** Perceptual self-motion thresholds for each plane of movement, obtained control subjects (*n* = 21) and patients with bilateral vestibulopathy (BV, *n* = 19), in the age group 60–79 years. Each box plot represents the 25–75 percentiles, whiskers indicate the 95 percentiles and bold black lines the median. Asterisks (*) illustrate statistically significant differences (*p* ≤ 0.001)

Median perceptual self-motion thresholds of BV patients were higher compared to control subjects for all type of movements in both age groups (40–59 years and 60–79 years), as illustrated in Fig. 1a and b. Thresholds were significantly higher in five out of six tested planes of movement in both age groups. Thresholds of roll rotations were not significantly different in the age group 40–59 years and thresholds of upward–downward translations were not significant in the age group 60–79 years (see Table 1 for a detailed overview of all median thresholds and Mann–Whitney *U* test statistics). Perceptual self-motion thresholds for translations varied widely within age groups for both control subjects and for patients with BV. Regarding rotations, perceptual self-motion thresholds showed less variability within age groups for control subjects, but not for patients with BV (Fig. 1a and 1b).

**Table 1:** Median thresholds (with interquartile range) for each direction of translation (m/s^2^) and rotation (°/s^2^), obtained in 34 control subjects and 37 patients with bilateral vestibulopathy (BV) split in age groups (40–59 years and 60–79 years)

Subjects	Translation Forward + backward	Translation Left + right	Translation Up + down	Yaw Left + right	Pitch Forward + backward	Roll Left + right
Age 40–59						
Control (*n* = 13)	0.10 (0.04)	0.10 (0.08)	0.14 (0.11)	1.50 (1.38)	0.20 (0.98)	0.10 (0.95)
BV (*n* = 18)	0.25 (0.17)	0.30 (0.20)	0.25 (0.15)	41.50 (17.75)	30.50 (33.49)	0.35 (43.03)
Mann–Whitney *U*	23.00	22.50	37.50	1.00	20.00	78.00
*p* value	0.000*	0.000*	0.001*	0.000*	0.000*	0.125
Age 60–79						
Control (*n* = 21)	0.14 (0.08)	0.14 (0.09)	0.20 (0.13)	1.50 (2.01)	0.30 (1.35)	0.20 (0.45)
BV (*n* = 19)	0.25 (0.19)	0.33 (0.26)	0.28 (0.19)	41.50 (34.00)	27.50 (38.50)	38.50 (43.95)
Mann–Whitney *U*	67.00	76.00	116.00	2.00	44.00	63.00
*p* value	0.000*	0.001*	0.023	0.000*	0.000*	0.000*

Asterisks indicate significant *p* values according to the Mann–Whitney *U* test with Bonferroni correction for multiple tests

### Patterns of self-motion perception performance and vestibular function using cluster analysis in BV patients

**Fig. 2:**
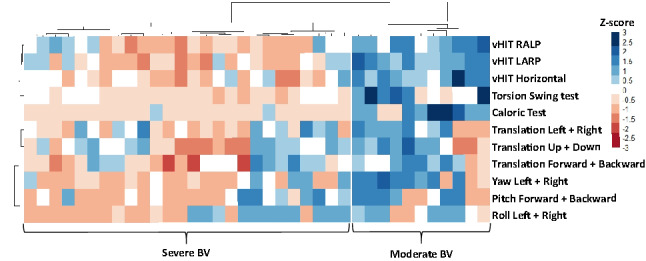
Heatmap as a result of hierarchical cluster analysis with two dendrograms; Each column represents one subject; each row represents the results of a specific vestibular test. A “bad (vestibular) score” (i.e. low scores on tests of vestibular reflexes and high thresholds on platform tests) is illustrated by lower *Z* scores in the color red. A “relatively good (vestibular) score” (i.e. relative high scores on tests of vestibular reflexes and low thresholds on platform tests) is illustrated by higher *Z* scores in the color blue. Curly brackets indicate the two clusters; “Severe BV” and “Moderate BV”. (*BV* Bilateral vestibulopathy, *vHIT* video head impulse test, *RALP* right-anterior–left-posterior, *LARP* left-anterior–right-posterior)

**Fig. 3:**
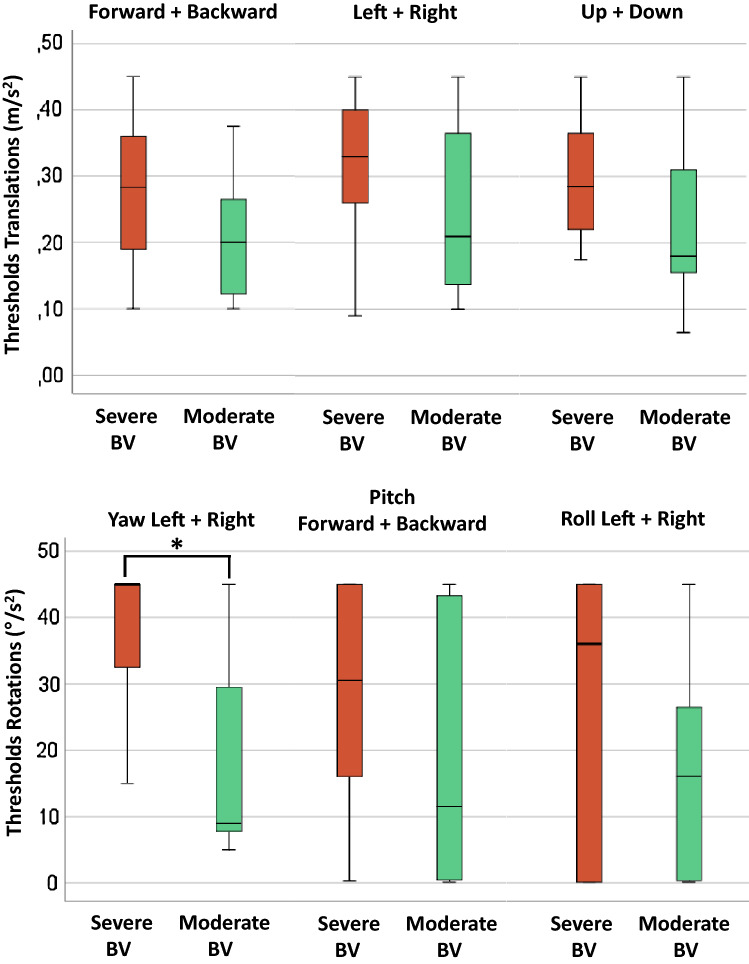
Perceptual self-motion thresholds for each plane of movement, obtained in 37 patients with BV split in two clusters (Severe BV; Moderate BV) as a result of hierarchical cluster analysis. Each box plot represents the 25–75 percentiles, whiskers indicate the 95 percentiles and bold black lines the median. The asterisk (*) illustrates a statistically significant difference (*p* ≤ 0.001). (*BV* Bilateral vestibulopathy)

**Fig. 4:**
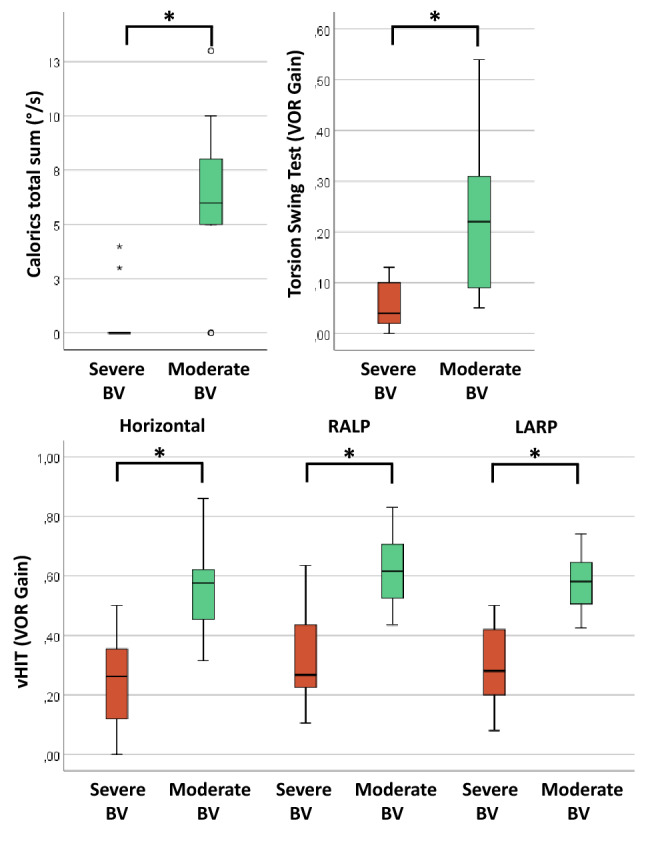
Results of tests of vestibular reflexes (caloric test, torsion swing test, vHIT), obtained in 37 patients with BV split in two clusters (Severe BV and Moderate BV) as a result of hierarchical cluster analysis. Each box plot represents the 25–75 percentiles, whiskers indicate the 95 percentiles and bold black lines the median. Asterisks (*) illustrate statistically significant differences (*p* ≤ 0.001). (*BV* bilateral vestibulopathy, *vHIT* video head impulse test, *RALP* right-anterior–left-posterior, *LARP* left-anterior–right-posterior)

In the group of BV patients, hierarchical cluster analysis resulted, according to the silhouette method [56], in two clusters with different patterns in perceptual self-motion thresholds and results of the other vestibular tests (Fig. 2). The first cluster (“Severe BV”; *n* = 26; 50% female; mean age 60 years) showed higher median perceptual self-motion thresholds for all planes of movement (Fig. 3), and lower median test results on all other vestibular tests (Fig. 4), compared to the second cluster (“Moderate BV”; *n* = 11; 45% female; mean age 62). These differences were significant between the clusters for perceptual self-motion thresholds in yaw plane, and results of the caloric test, torsion swing test and vHIT. Table 2 presents a detailed overview of all median thresholds, vestibular test results, and Mann–Whitney *U* test statistics.

**Table 2:** Median thresholds (with interquartile range) for age and each direction of translation (m/s^2^) and rotation (°/s^2^) and median vestibular test results (with interquartile range) for the caloric test (°/s), torsion swing test (VOR gain) and video head impulse test (vHIT) in the horizontal, right-anterior–left-posterior (RALP) and left-anterior–right-posterior (LARP) canal planes (VOR gain), obtained in 37 patients with bilateral vestibulopathy (Severe BV; Moderate BV)

	Age	Translation Forward + backward	Translation Left + right	Translation Up + down	Yaw Left + right	Pitch Forward + backward	Roll Left + Right	Calorics Total sum	Torsion 0.1 Hz	vHIT Horizontal	vHIT RALP	vHIT LARP
Severe BV (*n* = 26)	57.50 (12.50)	0.28 (0.18)	0.33 (0.16)	0.29 (0.15)	45.00 (12.88)	30.50 (29.46)	36.00 (44.90)	0.00 (0.00)	0.04 (0.08)	0.26 (0.24)	0.27 (0.22)	0.28 (0.22)
Moderate BV (*n* = 11)	65.00 (17.00)	0.20 (0.17)	0.21 (0.31)	0.18 (0.18)	9.00 (35.00)	11.50 (44.55)	16.05 (30.70)	6.00 (4.00)	0.22 (0.26)	0.58 (0.19)	0.62 (0.22)	0.58 (0.16)
Mann–Whitney *U*	121.00	90.50	100.00	87.00	50.00	91.50	123.50	28.00	38.00	13.00	22.00	9.00
*p* value	0.464	0.081	0.151	0.062	0.001*	0.087	0.523	0.000*	0.000*	0.000*	0.000*	0.000*

Asterisks indicate significant *p* values according to the Mann–Whitney *U* test with Bonferroni correction for multiple tests

Regarding VEMPs and perceptual self-motion thresholds, no association was found between present, present/absent or absent VEMPs and perceptual self-motion thresholds of translations. VEMP thresholds were above measurable threshold (i.e. no present p13n23 wave for cVEMP or n10p15 wave for oVEMP at 130 dB SPL) in 51% and 81% for cVEMP and oVEMP, respectively. VEMP thresholds were measurable in 24% and 8% for cVEMP and oVEMP, respectively. In nine patients (24%), cVEMP responses were present in one ear and absent in the other ear and in four patients (11%), oVEMP responses were present in one ear and absent in the other ear. A detailed overview of the Kendall’s tau-*b* test statistics can be found in Online Resource 3 Table 5.

## Discussion

This study showed that perceptual self-motion thresholds tested with the clinically oriented testing paradigm are significantly higher in BV patients compared to control subjects regarding nearly all rotations and translations. Next to this, cluster analysis showed that within the group of BV patients, higher perceptual self-motion thresholds are generally associated with lower residual vestibular function.

While these results are generally in line with previous research, this study offers three main contributions to the existing body of knowledge: (1) the use of a less elaborate and more clinically oriented testing paradigm, (2) the inclusion of a relatively large cohort of BV patients diagnosed according to the Bárány criteria and (3) the visualization of self-motion perception performance next to vestibular function in BV patients [3, 11, 17, 23, 28].

First, the use of a less elaborate and more clinically oriented testing paradigm results in differences related to the amount and type of motion-directions tested (i.e. a 12-option testing paradigm), the profile of the stimulus (i.e. stimuli with the longest possible exposition to constant peak acceleration) and the time of testing (i.e. a maximum duration of 1 h) [11, 23, 25]. This prevents the comparison of absolute thresholds between this study and previous literature. However, it would make the clinically oriented testing paradigm potentially easier to implement in future clinical practice.

Second, with regard to the study population as opposed to previous research, this study included a relatively large and heterogenous cohort of BV patients. Furthermore, previous studies reported self-motion perception performance of patients with vestibular hypofunction across a broad range of vestibular impairment (i.e. from having mild vestibular impairment to complete bilateral vestibular loss) [11, 23, 28], whereas the current study included patients according to diagnostic criteria as proposed by the Bárány Society [3], ensuring a defined range of vestibular impairment.

Despite differences in testing paradigms and study populations, both this study and existing literature found that (1) the vestibular system plays a crucial role in self-motion perception and (2) the extent to which the vestibular organs contribute to self-motion perception depends on the type of motion and the strategy of the central vestibular system to cope with the peripheral vestibular loss. Regarding the type of motion, especially during yaw rotations, the relative contribution of the vestibular organs is expected to be higher than during translations, pitches and rolls. After all, despite the correct precautions to minimize the somatosensory input, during translations or rotations, such as pitches and rolls, the center of mass is displaced or tilted with respect to gravity, respectively, increasing the somatosensory contribution [23, 25, 57]. It could be hypothesized that a greater somatosensory contribution in addition to vestibular input, might lead to a higher inter-subject variability in perceptual thresholds, since the somatosensory system is not as precise as the vestibular organs in detecting self-motion [58]. This could (partially) explain the higher inter control subject variability in perceptual thresholds for translations compared to rotations, and the higher variability in perceptual thresholds for rotations in the BV group compared to control subjects (Fig. 1a and 1b). Regarding the latter, this variability probably increased since BV patients had to rely more on their somatosensory system to detect self-motion [59].

The relative crucial role of vestibular input during yaw rotations was also shown by the third contribution of this study, namely the visualization of patterns of self-motion perception performance and vestibular function. Only perception of yaw rotations was significantly different between the two clusters “Severe BV” and “Moderate BV”, in which particularly the residual vestibular function differed (Fig. 3). It cannot be excluded that the diagnostic criteria of BV used for study inclusion (only measurements of the horizontal semicircular canals, involved in detecting yaw rotations) also played a role in this outcome. This seems however less likely, since also significant differences were found between the two clusters in vHIT results of the RALP and LARP planes. These planes of motion were not mainly tested during yaw rotations.

Additionally, this study showed no correlation between VEMPs and translations. This does not necessarily rule out a relation between VEMPs and perceptual self-motion performance for translations. After all, different factors can be identified that challenged evaluation of the correlation between VEMPs and perception of translations: the high inter-subject variability of VEMP responses, the somatosensory contribution during translations (leading to a high inter-subject variability in perceptual thresholds), measuring VEMP thresholds instead of VEMP amplitudes, and physical and physiological limitations (e.g. not stimulating louder than 130 dB SPL and not stimulating faster than 0.4 m/s^2^) which might have led to ceiling effects in VEMP and translational perceptual self-motion thresholds. Regarding the latter two arguments, a correlation between abnormal vibration-evoked oVEMPs amplitudes and higher perceptual self-motion thresholds of translations in the horizontal plane was shown by a previous study [29]. No significant correlation between perceptual self-motion thresholds and sound-evoked cVEMPs was found [29], but it was noted that a ceiling effect was observed. Next to this, it might be possible that categorizing VEMP data contributed to the inability to detect a correlation between perceptual self-motion thresholds of translations and VEMP thresholds. This is, however, considered to be less likely because of the small number of patients in the present and present/absent VEMP threshold categories.

Based on the findings of this study and previous research, it appears that vestibular function and somatosensory contribution are not the only factors contributing to self-motion perception. After all, studies investigating the effect of unilateral hypofunction on perceptual self-motion thresholds showed that thresholds were elevated and asymmetric at the expense of the affected side [60–62], and despite the vestibulo-ocular reflex becoming symmetric over time, self-motion thresholds remained significantly asymmetrically elevated [61]. These results suggest that central compensation might not be able to reach up to the initial state, compared with the vestibulo-ocular reflex [60, 61]. Similar to the results of this current study, it contributes to the understanding that vestibular organs provide information essential for self-motion perception, and nevertheless, it also depends on the strategy of the central vestibular system to cope with peripheral vestibular losses. Since self-motion perception is not only related to vestibular function, but also to other modalities such as central processing [18], it results in high inter-subject variability and, therefore, overlap between BV and control subjects. It could be hypothesized that this overlap is even larger for patients with unilateral vestibular hypofunction and for patients with bilaterally mild vestibular impairment. Therefore, it is expected to be a less appropriate tool for diagnostics.

Previously an association was shown between the etiology of BV and different deficits of the vestibular system [14, 32], suggesting that different etiologies might lead to differences in self-motion perception. For example, if an etiology would relatively “spare” the anterior semicircular canal, this might lead to relatively better self-motion perception in the plane of the anterior semicircular canal [32]. In this study presented here, etiology of BV was not taken into account. This was explicitly not done, since it would be preferred to have higher numbers of participants to reliably investigate the influence of etiology.

There are some methodological and equipment-related considerations for implementing self-motion perception testing in the clinical practice. Concerning methodology, extensive research-oriented protocols can be time-consuming [11, 20, 22–24], whereas clinical-oriented testing paradigms could be easier to implement in daily practice. Furthermore, a clinically oriented testing paradigm might be more applicable for patients with conditions affecting attention performance such as BV [27]. However, to evaluate self-motion perception of all translational and rotational movements, a six-degree-of-freedom motion platform is necessary, which is not commonly available [24]. Nonetheless, recent literature proposed that the total cost of equipment used in vestibular clinics today (e.g. caloric irrigator, video-nystagmography, electronystagmography, vHIT device, rotatory chair, VEMP equipment) would certainly exceed the cost of a single motion device [24]. Next to this, current tests used in the vestibular clinic primarily measure vestibular reflexes, whereas evaluation of self-motion perception provides information about input and central processing of peripheral vestibular and somatosensory signals.

This study helped giving direction to future research and potential settings in which determining perceptual self-motion thresholds can be of value. For implementation in research setting (and possibly clinical practice in the future), it is suggested to investigate self-motion perception as a functional outcome measure for vestibular rehabilitation (e.g. before and after vestibular implantation) and not as a diagnostic tool for vestibular disorders since self-motion perception is not only related to vestibular function. Regarding vestibular rehabilitation, previous studies showed that noisy galvanic vestibular stimulation improved self-motion perception in patients with BV [37, 45–47]. If the same trend is observed after vestibular implantation, it could be valuable to include testing perceptual self-motion thresholds as an outcome measure or even as part of the implantation criteria (e.g. for patients who have considerable complaints and abnormally elevated perceptual thresholds, but do not exactly meet implantation criteria) [63]. When self-motion perception is used as a functional outcome measure of vestibular rehabilitation, consider to include at least rotations in yaw plane. These might be the most sensitive representation of self-motion perception by the vestibular organs.

### Limitations

Due to the physical limitations of the platform, the maximum measurable acceleration was 0.4 m/s^2^ for translations and 40°/s^2^ for rotations. Therefore, patients who scored above the measurable threshold were rated with a threshold of 0.45 m/s^2^ for translations and 45°/s^2^ for rotations in this analysis. This could imply that the actual severity of the impaired vestibular perception was underestimated in this study, especially for yaw rotations, since 43% scored above the measurable threshold. Therefore, to determine the actual perceptual self-motion thresholds, the maximum measurable acceleration of the perception platform needs to extend the current maximum of 0.4 m/s^2^ for translations and 40°/s^2^ for rotations.

The actual vestibular function of subjects in the control group was not known since tests of vestibular reflexes were only performed in patients with BV. However, exclusion criteria for control subjects comprised current (or history of) vestibular disease; therefore, their vestibular function was expected to be within the range of normal. If vestibular deficits would have been present in some control volunteers, it would have mainly resulted in an underestimation of the findings in this study.

## Conclusion

Self-motion perception is significantly decreased in BV patients compared to control subjects regarding nearly all rotations and translations, tested with the clinically oriented testing paradigm. Furthermore, decreased self-motion perception is generally associated with lower residual vestibular function in BV patients. This is mainly seen in yaw rotations, probably due to the relatively lower somatosensory contribution compared to other motion profiles and directions. This might make rotations in yaw plane the most sensitive representation of self-motion perception by the vestibular organs. It is suggested to investigate self-motion perception as a functional outcome measure for vestibular implantation in research setting, and possibly clinical practice in the future.

## Supplementary Information

Below is the link to the electronic supplementary material.

Supplementary file1 (PDF 164 KB)

Supplementary file2 (PDF 1132 KB)

Supplementary file3 (PDF 130 KB)

## Data Availability

All included raw data is available upon request.
