# Supplementary material for: Bilateral vestibulopathy decreases self-motion perception
Source: J Neurol. 2021 Jul 14;269(10):5216–28. doi: 10.1007/s00415-021-10695-3 (PMC9467944; doi:10.1007/s00415-021-10695-3)
Supplement: Supplementary file 1 — Supplementary file1 (PDF 164 KB) [file 415_2021_10695_MOESM1_ESM.pdf]

Online Resource 1 – Platform details

Bilateral Vestibulopathy Decreases Self-Motion Perception

Lisa van Stiphout[1], Florence Lucieer[1], Maksim Pleshkov[1,2], Vincent van Rompaey[3], Josine Widdershoven[1,3], Nils Guinand[4], Angélica Pérez Fornos[4], Herman Kingma[1,2], and Raymond van de Berg[1,2]

1 Department of Otorhinolaryngology and Head and Neck Surgery, Division of Balance Disorders, Maastricht University Medical Center, School for Mental Health and Neuroscience, Maastricht, Netherlands
2 Faculty of Physics, Tomsk State Research University, Tomsk, Russian Federation
3 Department of Otorhinolaryngology and Head and Neck Surgery, Antwerp University Hospital, Faculty of Medicine and Health Sciences, University of Antwerp, Antwerp, Belgium.
4 Service of Otorhinolaryngology Head and Neck Surgery, Department of Clinical Neurosciences, Geneva University Hospitals, Geneva, Switzerland

Corresponding author: Lisa van Stiphout, lisa.van.stiphout@mumc.nl

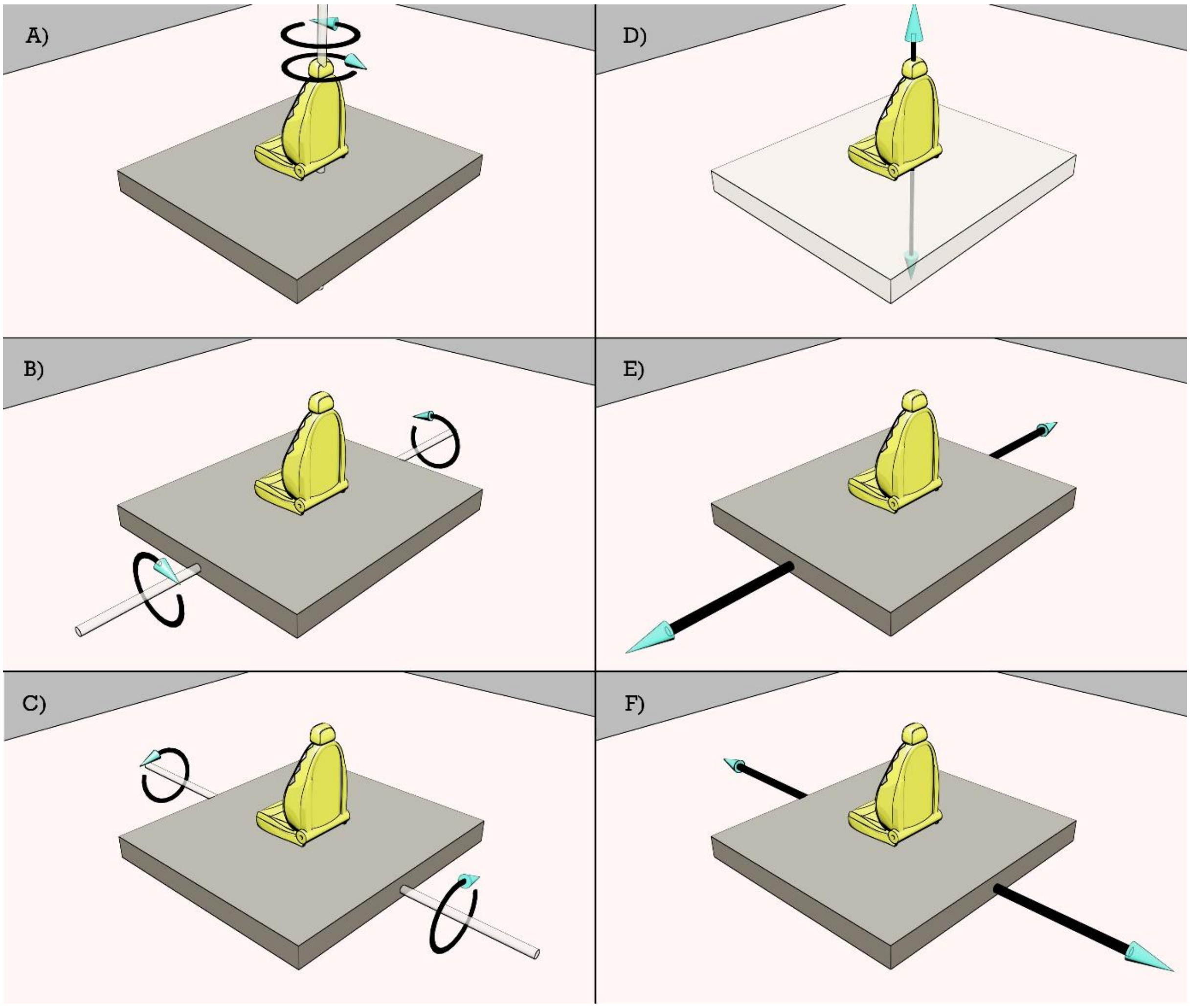

Figure 1. An overview of the twelve different motions and motion planes delivered by the platform (a hydraulic CAREN platform combined with D-flow 3.22.0 software (Motek Medical BV, Amsterdam, The Netherlands)). The platform was able to deliver six translations and six rotations. The six rotations included yaw left and yaw right (A), pitch forward and pitch backward (B), and roll left and roll right (C). The six translations included motions in the vertical plane: up and down (D), and in the horizontal plane: right and left (E), and forward and backward (F).
