# Supplementary material for: Bilateral vestibulopathy decreases self-motion perception
Source: J Neurol. 2021 Jul 14;269(10):5216–28. doi: 10.1007/s00415-021-10695-3 (PMC9467944; doi:10.1007/s00415-021-10695-3)
Supplement: Supplementary file 2 — Supplementary file2 (PDF 1132 KB) [file 415_2021_10695_MOESM2_ESM.pdf]

Online Resource 2 – Exemplar vestibular data

Bilateral Vestibulopathy Decreases Self-Motion Perception

Lisa van Stiphout[1], Florence Lucieer[1], Maksim Pleshkov[1,2], Vincent van Rompaey[3], Josine Widdershoven[1,3], Nils Guinand[4], Angélica Pérez Fornos[4], Herman Kingma[1,2], and Raymond van de Berg[1,2]

1 Department of Otorhinolaryngology and Head and Neck Surgery, Division of Balance Disorders, Maastricht University Medical Center, School for Mental Health and Neuroscience, Maastricht, Netherlands
2 Faculty of Physics, Tomsk State Research University, Tomsk, Russian Federation
3 Department of Otorhinolaryngology and Head and Neck Surgery, Antwerp University Hospital, Faculty of Medicine and Health Sciences, University of Antwerp, Antwerp, Belgium.
4 Service of Otorhinolaryngology Head and Neck Surgery, Department of Clinical Neurosciences, Geneva University Hospitals, Geneva, Switzerland

Corresponding author: Lisa van Stiphout, lisa.van.stiphout@mumc.nl

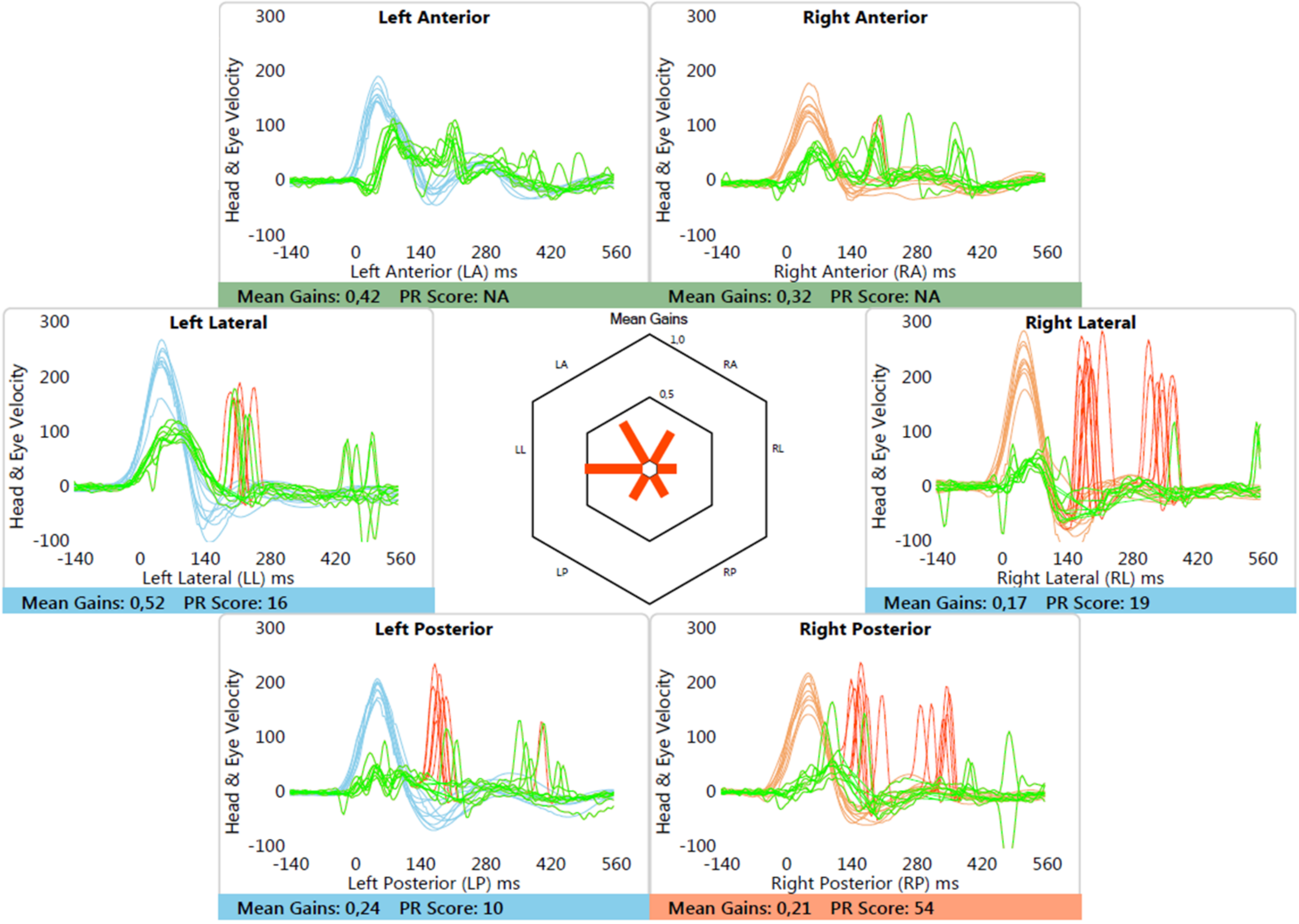

Figure 1. Exemplar video head impulse recordings and VOR gain (Otometrics, Taastrup, Denmark) of all semicircular canals in a patient with bilateral vestibulopathy.

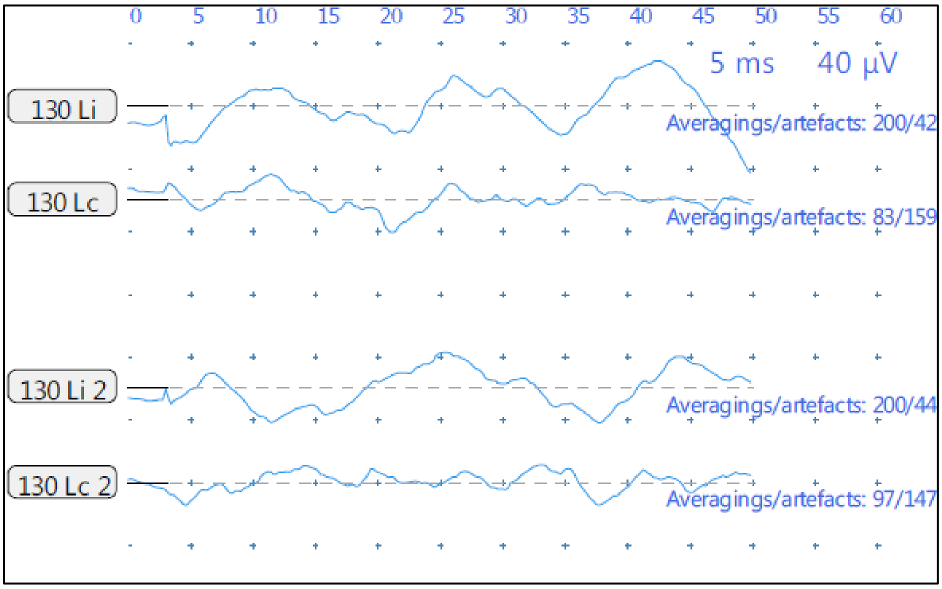

Figure 2. Exemplar left cervical vestibular evoked myogenic potential (cVEMP) wave form data from air-conducted tone bursts of 500Hz, provided via inserted earphones at a stimulation rate of 13Hz from a patient with bilateral vestibulopathy with no response at 130 dB SPL (Electromyographic software: Neuro-Audio, Difra, Belgium; self-adhesive electrodes: Blue sensor, Ambu, Denmark).

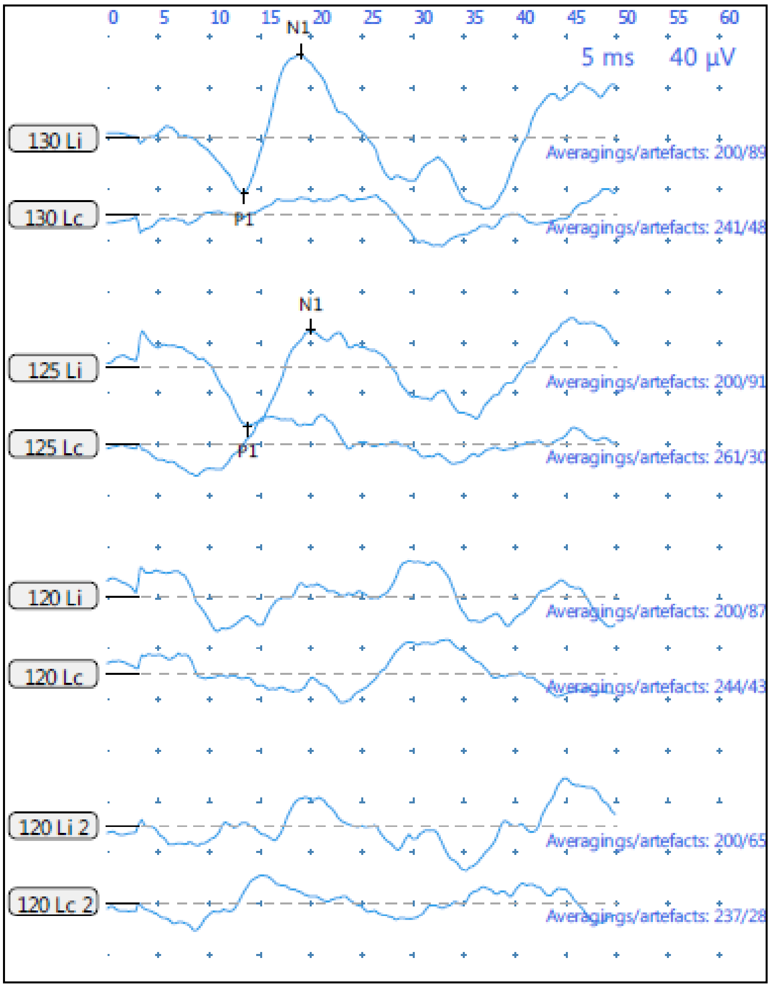

Figure 3. Exemplar left cervical vestibular evoked myogenic potential (cVEMP) wave form data from air-conducted tone bursts of 500Hz, provided via inserted earphones at a stimulation rate of 13Hz from a patient with bilateral vestibulopathy with a threshold at 125 dB SPL (Electromyographic software: Neuro-Audio, Difra, Belgium; self-adhesive electrodes: Blue sensor, Ambu, Denmark).

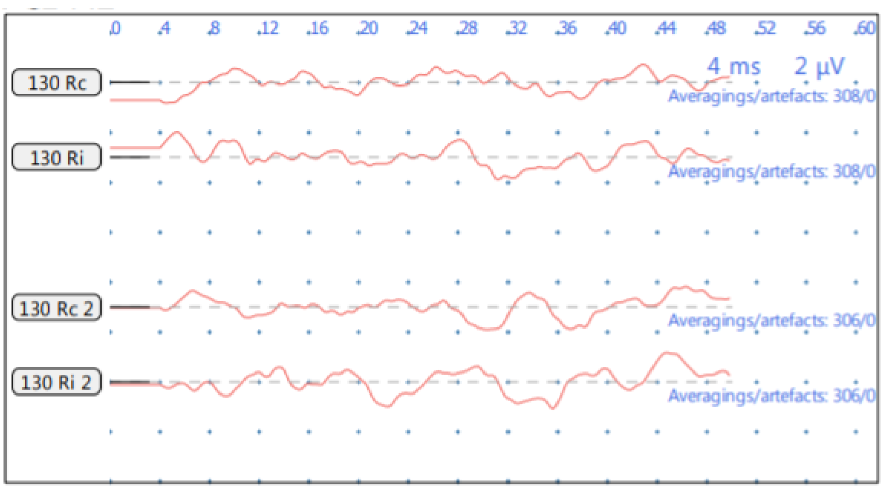

Figure 4. Exemplar right ocular vestibular evoked myogenic potential (oVEMP) wave form data from air-conducted tone bursts of 500Hz, provided via inserted earphones at a stimulation rate of 13Hz from a patient with bilateral vestibulopathy with no response at 130 dB SPL (Electromyographic software: Neuro-Audio, Difra, Belgium; self-adhesive electrodes: Blue sensor, Ambu, Denmark).

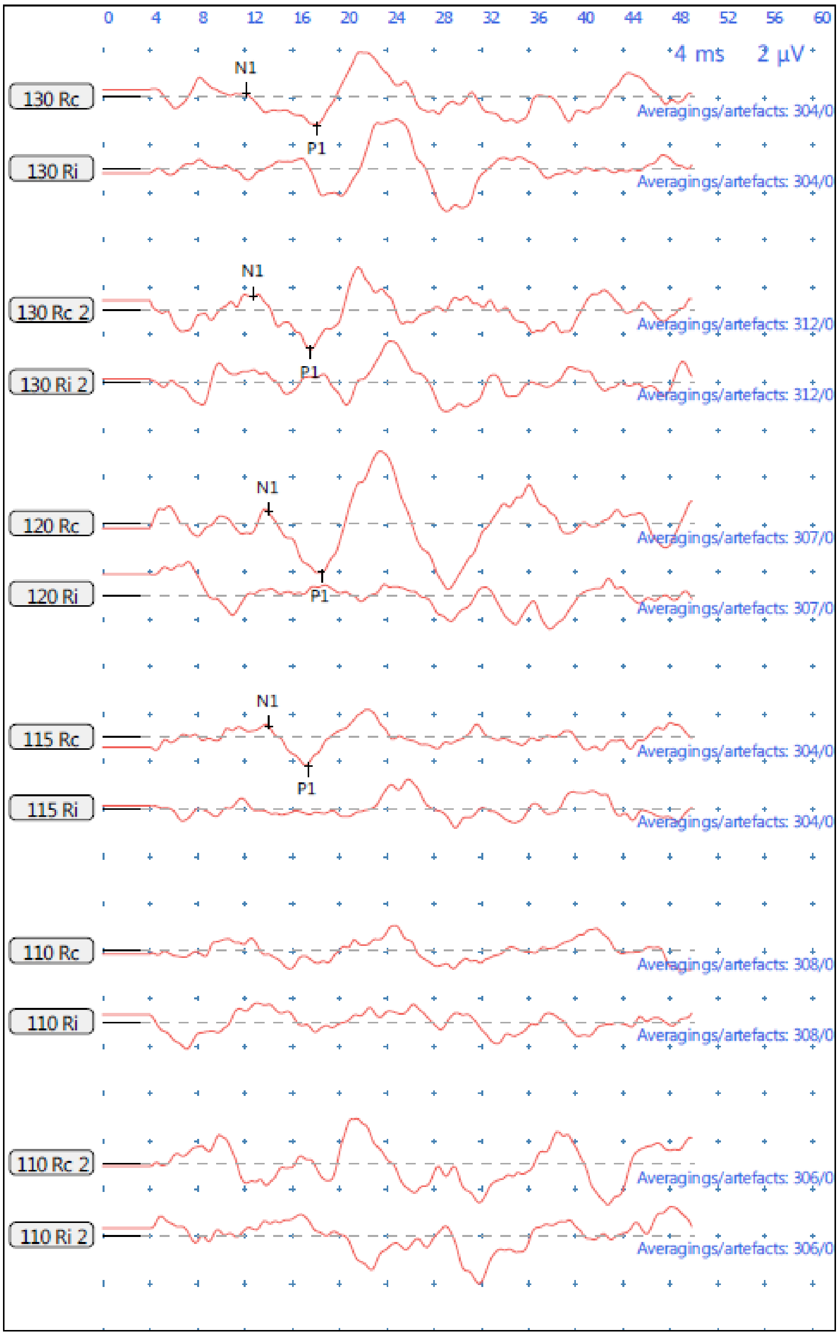

Figure 5. Exemplar right ocular vestibular evoked myogenic potential (oVEMP) wave form data from air-conducted tone bursts of 500Hz, provided via inserted earphones at a stimulation rate of 13Hz from a patient with bilateral vestibulopathy with a threshold at 115 dB SPL (Electromyographic software: Neuro-Audio, Difra, Belgium; self-adhesive electrodes: Blue sensor, Ambu, Denmark).

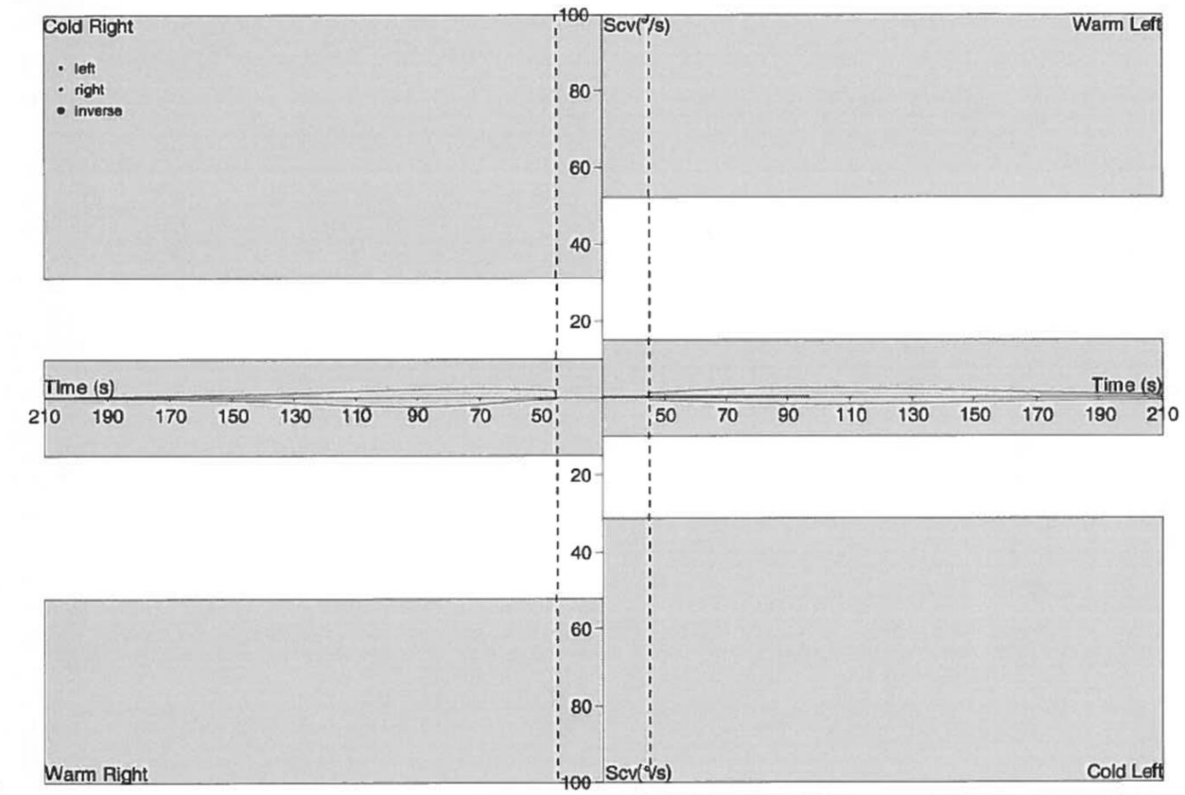

Nystagmus preponderance

SCV to the right 100% faster (< 25 %)

Side difference

Right side 100% less excitable (< 18 %)

	Amplitude of culmination phase	No. of nystagmus beats in culmination phase	Fixation supression	Spont. Nys.
**Warm Right :**	0 °/s (15,00 - 52,00 °/s)	0	---	0 R
**Warm Left :**	0 °/s (15,00 - 52,00 °/s)	0	---	0 R
**Cold Left :**	0 °/s (10,00 - 31,00 °/s)	0	---	0 R
**Cold Right :**	0 °/s (10,00 - 31,00 °/s)	0	---	0 R

Figure 6. Exemplar time course of the slow-phase velocity (SPV) of caloric nystagmus to warm (44°C) and cold (30°C) water external canal ear irrigation of the right and left ear from a patient with bilateral vestibulopathy. Eye movements were recorded with electronystagmography (KingsLab 1.8.1, Maastricht University, Maastricht, The Netherlands).

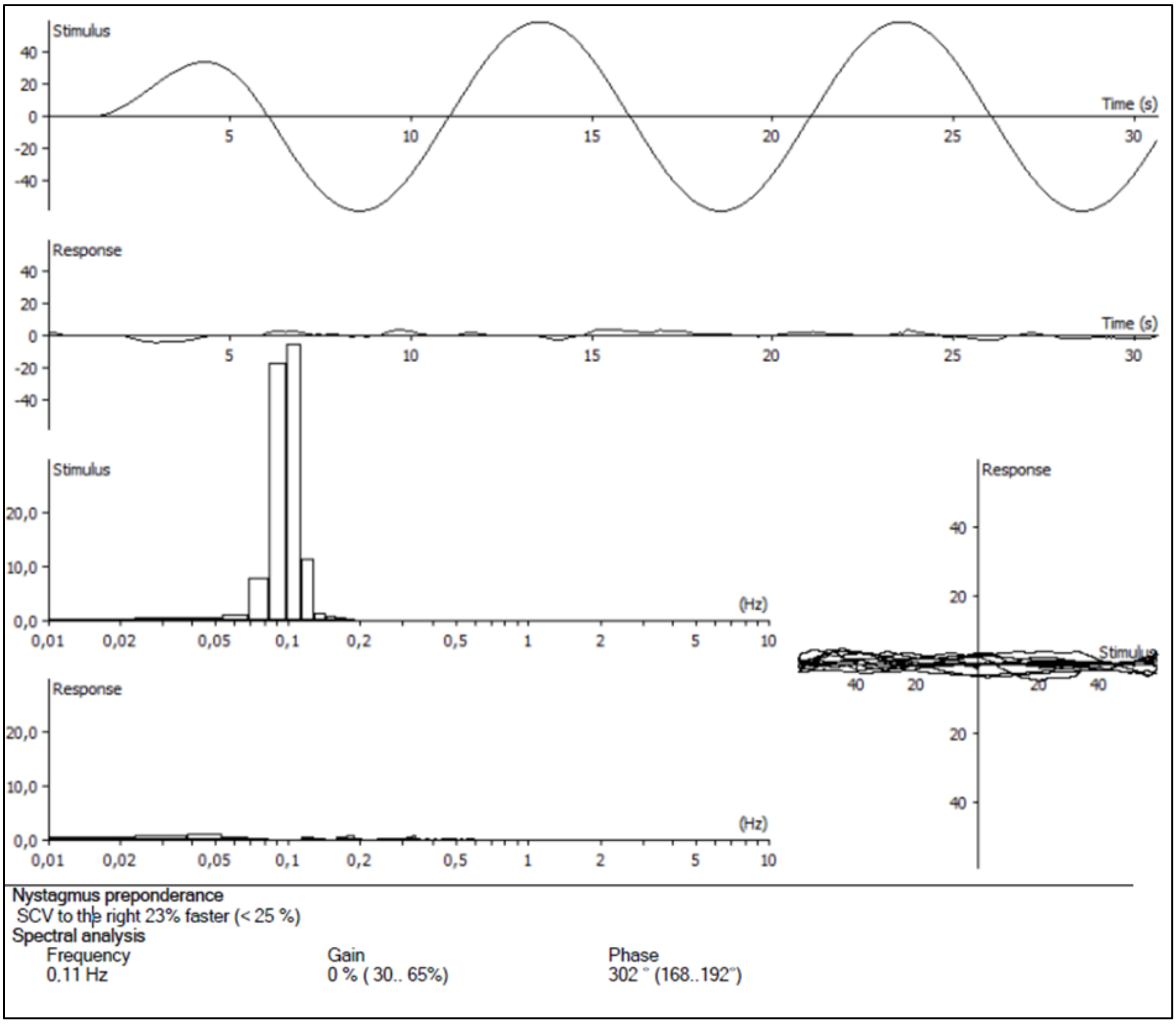

Figure 7. Torsion swing test recordings with a sinusoidal rotation (0.1Hz) and a peak velocity of 60°/s in a patient with bilateral vestibulopathy. Eye movements were recorded with electronystagmography (KingsLab 1.8.1, Maastricht University, Maastricht, The Netherlands).
