# Supplementary material for: Bilateral vestibulopathy decreases self-motion perception
Source: J Neurol. 2021 Jul 14;269(10):5216–28. doi: 10.1007/s00415-021-10695-3 (PMC9467944; doi:10.1007/s00415-021-10695-3)
Supplement: Supplementary file 3 — Supplementary file3 (PDF 130 KB) [file 415_2021_10695_MOESM3_ESM.pdf]

Online Resource 2 – Supplemental statistics and participant characteristics

Bilateral Vestibulopathy Decreases Self-Motion Perception

Lisa van Stiphout[1], Florence Lucieer[1], Maksim Pleshkov[1,2], Vincent van Rompaey[3], Josine Widdershoven[1,3], Nils Guinand[4], Angélica Pérez Fornos[4], Herman Kingma[1,2], and Raymond van de Berg[1,2]

1 Department of Otorhinolaryngology and Head and Neck Surgery, Division of Balance Disorders, Maastricht University Medical Center, School for Mental Health and Neuroscience, Maastricht, Netherlands
2 Faculty of Physics, Tomsk State Research University, Tomsk, Russian Federation
3 Department of Otorhinolaryngology and Head and Neck Surgery, Antwerp University Hospital, Faculty of Medicine and Health Sciences, University of Antwerp, Antwerp, Belgium.
4 Service of Otorhinolaryngology Head and Neck Surgery, Department of Clinical Neurosciences, Geneva University Hospitals, Geneva, Switzerland

Corresponding author: Lisa van Stiphout, lisa.van.stiphout@mumc.nl

Table 1. Median thresholds (with interquartile range) for each translation and rotation, obtained in 34 control subjects and 37 patients with bilateral vestibulopathy (BV). No significant differences were found between opposite movements according to the Mann-Whitney U test with Bonferroni correction for multiple tests, indicating opposite movements could be pooled.

	**BV (n=37)**	**Control (n=34)**
Translation Forward	0.22 (0.27)	0.12 (0.05)
Translation Backward	0.28 (0.22)	0.12 (0.08)
Mann-Whitney U	585.50	542.00
P-value	0.282	0.654
Translation Left	0.28 (0.27)	0.12 (0.13)
Translation Right	0.32 (0.28)	0.10 (0.08)
Mann-Whitney U	635.50	496.50
P-value	0.588	0.313
Translation Up	0.28 (0.25)	0.20 (0.14)
Translation Down	0.28 (0.23)	0.15 (0.12)
Mann-Whitney U	583.50	461.00
P-value	0.270	0.150
Yaw Left	45.00 (28.50)	1.50 (1.00)
Yaw Right	45.00 (31.50)	1.00 (1.88)
Mann-Whitney U	664.50	562.50
P-value	0.815	0.844
Pitch Forward	32.00 (44.35)	0.20 (0.90)
Pitch Backward	45.00 (38.50)	0.20 (0.90)
Mann-Whitney U	636.00	570.00
P-value	0.572	0.918
Roll Left	32.00 (44.90)	0.10 (0.20)
Roll Right	20.00 (44.90)	0.10 (0.40)
Mann-Whitney U	653.50	575.00
P-value	0.725	0.966

Table 2. Median thresholds (with interquartile range) for each translation and rotation, obtained in 37 patients with bilateral vestibulopathy (BV), split into two age categories. No significant differences were found between age categories according to the Mann-Whitney U test with Bonferroni correction for multiple tests.

**Subjects**	**Translation Forward + Backward**	**Translation Left + Right**	**Translation Up + Down**	**Yaw Left + Right**	**Pitch Forward + Backward**	**Roll Left + Right**
Age 40-59 (n = 18)	0.25 (0.17)	0.30 (0.20)	0.25 (0.15)	41.50 (17.75)	30.50 (33.49)	0.35 (43.03)
Age 60-79 (n =19)	0.25 (0.19)	0.33 (0.26)	0.28 (0.19)	41.50 (34.00)	27.50 (38.50)	38.50 (43.95)
Mann-Whitney U	154.50	160.00	146.50	154.00	164.50	116.50
P-value	0.620	0.753	0.461	0.620	0.845	0.098

Table 3. Relevant bilateral vestibulopathy patient group and control group characteristics (n=37)

	BV (n=37)	Control (n=34)
Age (mean, SD)	60 (9.7)	61 (10.3)
Female (%)	48.6	61.8
Male (%)	51.4	38.2

Table 4. Relevant bilateral vestibulopathy patient group characteristics (n=37)

**BV-patient group characteristic**	**Percentage (%)**
**Etiology**	
Definite etiology	54
Ototoxic	40
Infectious	25
Genetic	20
Other	15
Etiology idiopathic or of probable cause	46
**Vestibular test results**	
Bilateral reduced caloric response (sum bithermal SPV <6°/s)	89
Bilateral reduced VOR gain measured with vHIT (<0.6)	84
Reduced VOR gain on torsion swing test (< 0.1)	60
**BV severity according to the number of met Bárány criteria**	
Three out of three	54
Two out of three	24
One out of three	22

Table 5. The relationship between present, present/absent or absent ocular and cervical Vestibular Evoked Myogenic Potentials (oVEMP and cVEMP) and perceptual self-motion thresholds of translations, assessed with Kendall's tau-b correlation. No statistically significant association was found.

		**oVEMP**	**cVEMP**
**Translation Forward + Backward**	Correlation Coefficient	-0.07	-0.14
	P-value	0.59	0.28
**Translation Left + Right**	Correlation Coefficient	0.19	-0.10
	P-value	0.17	0.47
**Translation Up + Down**	Correlation Coefficient	0.12	-0.13
	P-value	0.37	0.31
